# Impact of Procyanidins from Different Berries on Caspase 8 Activation in Colon Cancer

**DOI:** 10.1155/2015/154164

**Published:** 2015-06-09

**Authors:** Carole Minker, Livine Duban, Daniel Karas, Päivi Järvinen, Annelise Lobstein, Christian D. Muller

**Affiliations:** ^1^Laboratoire d'Innovation Thérapeutique, UMR 7200, Faculté de Pharmacie, Université de Strasbourg, 74 route du Rhin, BP 24, 67401 Illkirch Cedex, France; ^2^Merck Millipore Bioscience Division, 78180 Saint-Quentin-en-Yvelines, France; ^3^Department of Medical Chemistry and Biochemistry, Palacký University, Hněvotínská 3, 775 15 Olomouc, Czech Republic; ^4^UMR 7200 CNRS, Faculté de Pharmacie, 75 route du Rhin, 67400 Illkirch, France

## Abstract

*Scope*. The aim of this work is to identify which proapoptotic pathway is induced in human colon cancer cell lines, in contact with proanthocyanidins extracted from various berries. *Methods and Results*. Proanthocyanidins (Pcys) extracted from 11 berry species are monitored for proapoptotic activities on two related human colon cancer cell lines: SW480-TRAIL-sensitive and SW620-TRAIL-resistant. Apoptosis induction is monitored by cell surface phosphatidylserine (PS) detection. Lowbush blueberry extract triggers the strongest activity. When tested on the human monocytic cell line THP-1, blueberry Pcys are less effective for PS externalisation and DNA fragmentation is absent, highlighting a specificity of apoptosis induction in gut cells. In Pcys-treated gut cell lines, caspase 8 (apoptosis extrinsic pathway) but not caspase 9 (apoptosis intrinsic pathway) is activated after 3 hours through P38 phosphorylation (90 min), emphasizing the potency of lowbush blueberry Pcys to eradicate gut TRAIL-resistant cancer cells. *Conclusion*. We highlight here that berries Pcys, especially lowbush blueberry Pcys, are of putative interest for nutritional chemoprevention of colorectal cancer in view of their apoptosis induction in a human colorectal cancer cell lines.

## 1. Introduction

Colorectal cancer is presently the 3rd more diagnosed cancer in the world (1 million cases), after lung (1,35 million cases) and breast cancers (1,15 million cases) [1]. Concerning the worldwide incidence, colorectal cancer is placed at the 4th rank for men and at the 3rd rank for women. Epidemiologic studies indicate that physical inactivity and excess body weight as abdominal fat are risk factors to develop a colorectal cancer, in particular as a result of an unbalanced diet (high in saturated fats, low in vegetables and fruits) [2]. It is currently admitted that 60 to 80% of colorectal cancers could be avoided by a diet modification [3–5]. Colon carcinogenesis is a process which takes place on 10 to 20 years [6–8], representing a rather long period of time on a human life scale and accounting for the late average diagnosis age. This is the reason why chemoprevention is a particularly relevant strategy in the context of colorectal cancer. Chemoprevention, first described by Sporn in 1976, consists in the administration of natural, synthetic, or biochemical compounds able to prevent cancer apparition or suppress or even reverse its progression and extension [9, 10]. In the case of colorectal cancer, primary chemoprevention concerns the whole population with an average risk to develop this kind of cancer. Secondary chemoprevention is much aimed for a population with a high risk to develop a colorectal cancer [6, 11].

Nutritional cancer chemoprevention consists in* per os* administration of bioactive dietary compounds [12], presenting anticancer activities via different and complementary mechanisms of action [13, 14]. Bioactive dietary phytoconstituents are thus able to exert their activities on precancerous and cancerous colorectal cells at low but regular doses, such as a metronomic chemotherapy approach [15]. Strategies consisting in preventing and even treating cancer with natural dietary compounds able to induce apoptosis of cancerous cells are currently largely admitted and studied [16–21].

Proanthocyanidins (Pcys), also known as condensed tannins, are the most widely represented products of plants secondary metabolism throughout nature, after lignins [22, 23]. They are constituted by the assembly of flavan-3-ol monomer units, giving rise to Pcy oligomers (2 to 10 monomer units) and Pcy polymers (>10 units, up to 200) [24, 25]. These monomer units are most frequently epicatechin, epiafzelechin, and epigallocatechin, forming procyanidins, propelargonidins, and prodelphinidins, respectively [22]. Flavan-3-ol units can be linked by 2 types of bounds [22, 23, 25]: type B link, mostly C4→C8, or less frequent type A link consisting in a double bounding, for example, C4→C8 and C2→O→C7. The more widespread Pcys are procyanidins and heterogeneous combinations of different monomer units [22]. Pcys protect plants against external aggressions like UV, bacteria, fungi, insects, and herbivores [26, 27], as they are present in certain fruits, nuts, spices, and beverages [28, 29]. Pcys represent a large part of phytoconstituents in a balanced diet [30], and thus they can exert a wide variety of beneficial biological effects [28, 31]. While their* in vivo* antioxidant [32], anti-inflammatory [21], and vasculoprotective [33] activities have already been demonstrated, they are also currently studied for their beneficial effects against cancer, at different stages of its evolution [34].

Earlier, we demonstrated the* in vitro* and* in vivo* colon chemopreventive activities of apple Pcys [35–37]. We postulate here that Pcys from other natural edible sources may exert beneficial anticancer effects as well. Anticancer properties of lowbush blueberry (*Vaccinium myrtillus*) in colorectal cancer remain focused on their anthocyanins effective* in vitro* [38, 39] and* in vivo* [40]. Despite the growing interest on anticancer activities of Pcys [34], there is nowadays no study on proapoptotic activities on colorectal cell lines of lowbush blueberry Pcys. Therefore, we screened for proapoptotic activities of different Pcy-rich fractions obtained from various local fruits. Proapoptotic activities on a validated cellular model of colon cancer progression from a primary tumor were tested on SW480 a TRAIL-sensitive cell line [41] and its corresponding metastatic TRAIL-resistant SW620 sister cell line [42]. We finally focused on* Vaccinium myrtillus* berries, whose Pcys were found to be the most active, trying to clarify elements of their proapoptotic mechanism of action.

## 2. Materials and Methods

### 2.1. Fruit Extraction and Proanthocyanidin Enrichment

The following berries were obtained from a local organic producer “Les Fruits d'Altitude” (Fresse-sur-Moselle, France): wild lowbush blueberry (*Vaccinium myrtillus*), highbush blueberry (*Vaccinium corymbosum*), lingonberry (*Vaccinium vitis-idaea*), raspberry (*Rubus idaeus*), wild blackberry (*Rubus fruticosus*), thornfree blackberry (*Rubus fruticosus* “Thornfree”), redcurrant (*Ribes rubrum*), gooseberry (*Ribes uva-crispa*), blackcurrant (*Ribes nigrum*), and jostaberry (*Ribes nidigrolaria*). Cranberry (*Vaccinium macrocarpon*) was purchased from Ocean Spray (Canada).

Fresh fruits were extracted with acetone/water 6 : 4 (1 L/kg) for 3 × 24 h stirring, away from light. The crude extract was then dried and then taken up in water and extracted successively with cyclohexane and ethyl acetate. The aqueous extract was centrifuged to remove insoluble polymers of high MW and then fractionated on Sephadex LH-20 with 100% water followed by addition of 10% methanol with 10% (100 mL) and then washed with acetone/6/4 water (500 mL). Fractions were grouped according to their chromatographic profile (proanthocyanidins and degree of polymerization of greater than 3) and proapoptotic activity. Dosage of proanthocyanidins by vanillin showed that the enriched blueberry extract contains 101 ± 1 g catechin equivalents per 100 g of extract. DMAC proanthocyanidins dosage showed that enriched blackberry extract contains 32.5 g ± 5.1 g of procyanidin A2 equivalent to 100 g of extract. Pcy-rich extracts were obtained by mixing several of the above-cited fractions.

### 2.2. Proanthocyanidin Determination

DMAC (*p*-dimethylaminocinnamaldehyde) Pcy dosage generates more stable and reproducible results than vanillin [43]. In acidic conditions, DMAC specifically reacts with* meta*-diphenols to form a green carbonium ion detected at 640 nm. The DMAC dosage is as highly specific to Pcys as it does not react with other flavonoids like anthocyanins [44]. However, the colour development depends on the procyanidin structural conformation, and although it has not been demonstrated yet, several authors have suggested that the DMAC reagent could react with only one flavan-3-ol monomer inside a Pcys [45, 46] leading us to an underestimation of Pcy contents, especially for polymers. Procyanidin A2 was our internal standard, as recently validated by several laboratories for cranberry Pcy dosage [47]. Nevertheless, as apple (which we used as our internal standard) contains only type B Pcys, we checked the similarity to procyanidin B2 an internal standard recently published [48]. We observed similar results with both procyanidins A2 and B2 as internal standards.

### 2.3. Cells

SW480 is a cell line derived from a grade B primary colon carcinoma (Duke's classification) of a 50-year-old patient. SW620 cell line is derived from a metastasis located in a lymphatic node of the same patient, which is removed 6 months later. Both cell lines are obtained through European Collection of Animal Cell Culture (ECACC, Salisbury, UK). They are cultured in Alpha modified Eagle's medium supplemented by 10% heat-inactivated (56°C/30 min) fetal calf serum, 1% penicillin/streptomycin (10 000 U and 20 mg/mL), and 1% L-glutamine (PAN Biotech GmbH, Aidenbach, Germany). Incubations were carried out at 37°C in a humidified atmosphere with 5% CO_2_. The culture medium was replaced every 48 h. Cells were detached by 5 mL trypsin/EDTA (0.05%/0.02% in PBS) (PAN Biotech GmbH, Aidenbach, Germany). All experiments were carried out during exponential phase cell growth. THP-1 (TIB-202) and BxPC-3 (CRL-1687) cells were grown in RPMI 1640 medium with 2 mM L-alanyl-L-glutamine additionally supplemented with 10% (v/v) fetal bovine serum and 50 U/mL penicillin and 50 *μ*g/mL streptomycin (Sigma-Aldrich). HepG2 (HB-8065) cell line was maintained in MEM media supplemented with 10% (v/v) fetal bovine serum, 50 U/mL penicillin and 50 *μ*g/mL streptomycin (Sigma-Aldrich), and 2 mM glutamine. Cells were grown in humidified atmosphere with 5% CO_2_ at 37°C in 25 cm^2^ and 75 cm^2^ flasks up to 70–80% confluency prior to treatment.

Cells were incubated with the different compounds (Pcy extracts and/or TRAIL) while seeding and incubated for 24 h (SW620) or 48 h (SW480). For control, ethanol used to dissolve Pcy samples was added to the cells at a final concentration of 0.25% (v/v). Apple procyanidins (apple Pcy) used as a Pcy internal control are obtained from Applephenon extract (Maypro Industries, NY, USA) [35].

### 2.4. Apoptosis

Early and late apoptosis are monitored by flow cytometry (Guava PCA-96 Merck/Millipore, Molsheim, France). Late apoptotic cells are double labeled by Annexin V and 7-AAD (Guava Nexin Reagent kit Merck/Millipore). Apoptotic positive control for each experiment is obtained by celastrol (50 *μ*M) dissolved in DMSO [49, 50]. Celastrol, a gift from Pr. A. C. Allison (Alavita Pharmaceuticals Inc., CA USA), validated each experimental 96-well plate with a 90 to 100% observed rate of apoptosis.

Recombinant human TRAIL was purchased from R&D Systems Europe (Abingdon, UK). TRAIL was dissolved in DPBS supplemented by 1% fetal calf serum and tested at concentrations ranging from 0 to 100 ng/mL.

### 2.5. Cell Cycle Phase Distribution Analysis and Quantitation of Hypodiploid Sub-G1 Cell Population

Cells were cultured in 25 or 75 cm^2^ culture flasks at a density of 10^5^ up to 10^6^ cells/mL depending on the cell line in accordance with ATCC recommendations. After seeding for 24 h cells were exposed to the extracts for different time periods. Then cells were washed with phosphate-buffered saline (PBS), resuspended in ethanol 70%, and placed for 24 h at −20°C. After centrifugation at 400 g for 5 min, cells were washed twice with PBS buffer. Cells were then resuspended in 500 *μ*L PBS, incubated in FxCycle PI/RNase Staining Solution (Life technologies, Thermo Fisher Scientific Inc., USA), and kept in the dark at room temperature for 30 min. Cellular DNA content was then assessed by flow cytometry in a Guava EasyCyte Plus HP system (EMD Millipore Corporation, Billerica, MA, USA). A minimum of 10,000 cells were acquired per sample and analyzed on the InCyte software. The percentage of cells in G0/G1, S, G2/M, and sub-G1 was determined from DNA content histograms.

### 2.6. Death Receptors

TRAIL-R1 (DR4/CD261), TRAIL-R2 (DR5/CD262), and Fas (CD95/APO1) specific fluorescent antibodies were used to monitor their expressions onto the cell surface. TRAIL-R1 was labeled by an anti-human mouse monoclonal anti-TRAIL-R1 antibody coupled to Alexa Fluor 488 (AbD Serotec, Düsseldorf, Germany). TRAIL-R2 was revealed by an anti-human mouse monoclonal anti-TRAIL-R2 antibody coupled to phycoerythrin (PE) from CliniSciences (Montrouge, France). Fas was labeled by an anti-human mouse monoclonal anti-Fas antibody coupled to phycoerythrin-Cy5 (PE-Cy5) as well from CliniSciences (Montrouge, France).

After 24 h (SW620 cell line) or 48 h (SW480 cell line) incubation with Pcys, cells were centrifuged 5 min at 200 g and 4°C. Used medium was replaced by culture medium supplemented with antibody solutions. Cells were then incubated 3-4 h at 0°C protected from light. After incubation, cells were centrifuged again, and medium was replaced by fresh culture medium. Cells were then analysed by flow cytometry, on a Guava EasyCyte Plus device for TRAIL-R1 and TRAIL-R2 detection or on a Guava PCA-96 device for Fas detection. The excitation laser wavelength of the Guava EasyCyte Plus is 488 nm, whereas it is 525 nm for the Guava PCA-96.

### 2.7. Caspases 8 and 9

Caspases 8 and 9 activation was assessed by marking cells with Guava Caspase kit (Merck/Millipore, Molsheim, France). After 24 h (SW620 cell line) or 48 h (SW480 cell line) incubation with Pcys, cells were washed with PBS. Caspases were marked with FLICA reagents covalently marked with a fluorescent probe: FAM (6-fluorescein amidite) for caspase 8 and SR for caspase 9. Cells were incubated for 1 h at 37°C. They were then washed twice with PBS, marked with 7-AAD, and analyzed by flow cytometry on the Guava EasyCyte 8HT device. The Guava EasyCyte 8HT is equipped with two lasers whose excitation wavelengths are 488 and 640 nm.

### 2.8. ATF2 and P38

Measuring the activity of cell signaling pathways (ATF2 and P38) by flow cytometry was done with the FlowCellect p38 Stress Pathway Activation Detection Kit (Merck/Millipore, Molsheim, France). The anti-pP38 (Thr180/Tyr182) antibody is Alexa Fluor 488 labeled and the anti-TF2 (Thr69/71) antibody is tagged with Alexa Fluor 647. Cell staining protocol was done according to the manufactured recommendations and was analyzed on the Guava EasyCyte 8HT device.

### 2.9. Statistics

Data are reported as mean ± standard deviation of the mean (SD). Statistical analyses were evaluated using Student's* t*-test. For the preliminary screening on the thirteen fruits Pcy-rich fractions, values were corrected by Bonferroni's multiple comparison (threshold = 7.6 · 10^−4^).

Generally, ^*^ *P* < 0.05; ^**^ *P* < 0.01; ^***^ *P* < 0.001.

EC_50_ (effective concentration, 50%) determinations with sigmoidal dose-response were computed using GraphPad Prism version 5.0f for OSX (GraphPad Software, San Diego, California USA, http://www.graphpad.com/).

## 3. Results

### 3.1. Fruit Extraction and Proanthocyanidin Enrichment Yield

Twelve locally grown fruits were extracted and then fractioned as described in order to obtain several Pcy-rich fractions per fruit (Table 1).

### 3.2. Screening of Proapoptotic Activities of Pcy-Rich Fractions from Various Berries

**Figure 1:**
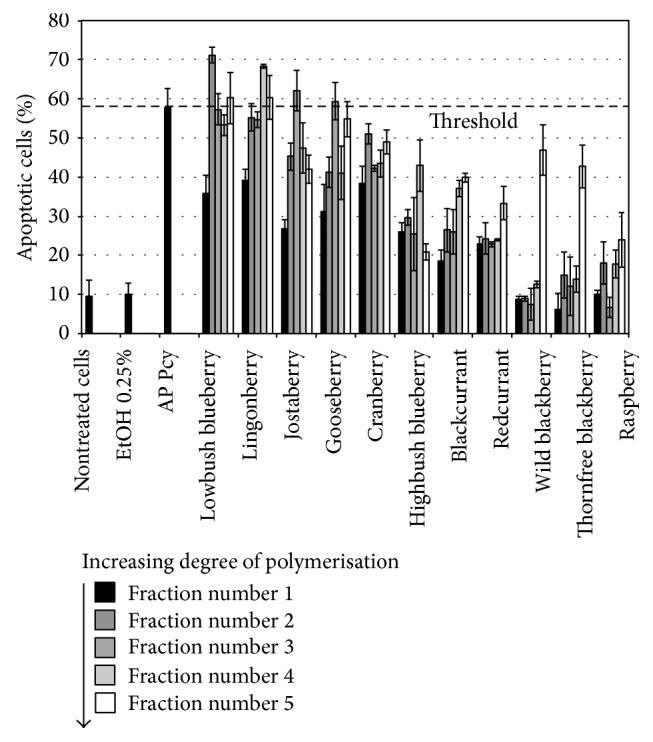
Proapoptotic activities. The 55 proanthocyanidin-rich fractions obtained from 11 fruits (5 fractions per fruit) were tested at 50 *μ*g/mL (final concentration) on SW620 cells for their apoptosis induction properties after 24 h of incubation. Apoptosis yield was evaluated in cytometry by PS cell surface expression as described under Materials and Methods.

The obtained fractions were then evaluated for proapoptotic activities on SW620 cells (Figure 1) and compared to apple procyanidins (apple Pcy), a standard well described [35–37].

Pcys from two locally grown fruits showed stronger proapoptotic activities than apple Pcy so that lowbush blueberry and lingonberry were chosen for further investigations on their proapoptotic activities on both SW620 and SW480 cell lines.

### 3.3. Proapoptotic Activities of Pcys from Lowbush Blueberry and Lingonberry on SW480 and SW620 Cell Lines

Pcy-rich extracts from lowbush blueberry and lingonberry were obtained by combining Pcy-rich fractions no. 2 to no. 5 from each fruit, respectively. Their Pcy richness was assessed by BL-DMAC dosage that estimates procyanidin A2 equivalents contents and then compared to apple Pcy (Table 1).

**Figure 2:**
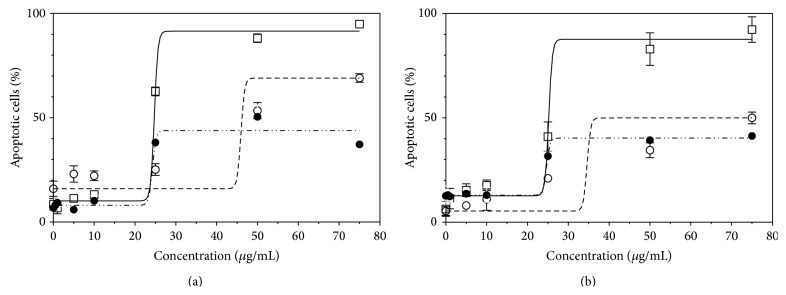
EC_50_ of proapoptotic activities. Dose effect curves were plotted for apple Pcy (- - -), proanthocyanidin-rich extracts obtained from lowbush blueberry (—), and lingonberry (··-··-) when tested on SW620 (a) or SW480 (b) cell lines.

These extracts were tested for their proapoptotic activities on SW480 and SW620 cell lines at several concentrations. Dose-response curves were computed to obtain EC_50_ values (Figure 2) with noteworthy differences between cell lines or Pcy origins. One should notice that only lowbush blueberry Pcys were able to induce more than 90% of apoptosis on both cell lines (92–95%), followed by apple Pcys (55–64%) and finally by lingonberry Pcys (37–41%) as shown in Table 2.

No clear correlation between activity and procyanidin A2 equivalents content could be observed.

Lowbush blueberry Pcys were selected as most active to carry out a more fine mechanistic approach.

### 3.4. Lowbush Blueberry Pcys Activities on the Human THP-1 Monocytic Cell Line

**Figure 3:**
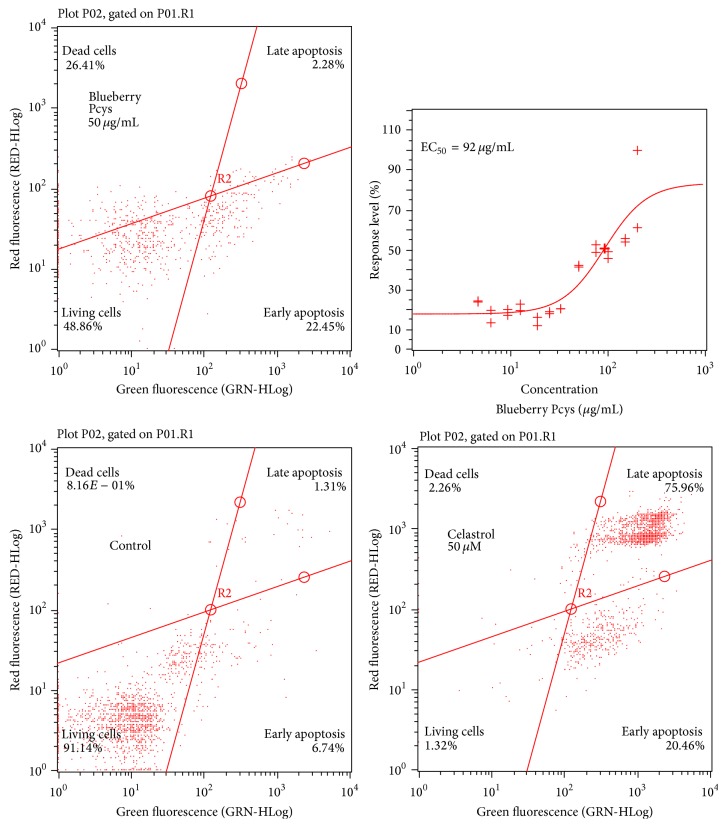
PS exposure on THP-1 monocytic cells induced by lowbush blueberry Pcys. Dose-response curve and EC_50_ value for lowbush blueberry Pcys incubated in raising concentrations were determined on THP-1 cells (upper right graph) after 24 h of incubation. Cells were stained using Annexin V-/PI apoptotic assay as described in Section 2.3 (*n* = 3 independent experiments; 2,000 events per sample were analyzed). Cytograms presented here illustrate the fact that Pcys on early apoptosis could be observed on the contrary of the data obtained for 50 *μ*M celastrol (lower right cytogram).

At around 100 *μ*g/mL Pcys, 50% of the cells exhibited PS on the cell surface (early apoptosis) when at the same time (Figure 3) celastrol (50 *μ*M) induced 97% of cells in late apoptosis. At 100 *μ*g/mL of fruit Pcys, Chacón et al. reported a percentage of viability >97% when monitoring LDH [51]. In the same manner after 24H of treatment, no increase in number of hypodiploid sub-G1 cells could be observed when monocytic cells THP-1 were exposed to 50 *μ*g/mL of lowbush blueberry Pcys (Table 3) suggesting no apoptotic activity in these cells. Taken together these data suggest an important cell surface disturbant activity of Pcys on THP-1 inflammatory cells without leading to an apoptotic cell death.

Fruit Pcys were reported to possess multiple biological activities including anti-inflammatory [52]. Fruit Pcys decrease production of inflammatory cytokines (at 100 *μ*g/mL), tumor necrosis factor-alpha, and interleukin-6, in cultured human monocytic THP-1 cells, in response to lipopolysaccharide [53]. It appears that, for THP-1 cells, Pcys exhibit a protective action on DNA degradation with mainly anti-inflammatory actions.

### 3.5. Lowbush Blueberry Pcys Are Not Able to Sensitize SW480 and SW620 Cell Lines to TRAIL-Induced Apoptosis

We described previously that apple Pcys were able to sensitize SW620 and SW480 cell lines to TRAIL-induced apoptosis [54]; we wanted to investigate whether lowbush blueberry Pcys could as well potentiate TRAIL-induced apoptosis of SW480 cell line and sensitize TRAIL-induced apoptosis on SW620 cell line.

A combination of increasing TRAIL and lowbush blueberry Pcy concentrations was tested on both cell lines with no proapoptotic synergistic effect (Figure 3) but lowbush blueberry Pcys are not able to sensitize neither SW480 nor SW620 cell lines to TRAIL-induced apoptosis.

### 3.6. TRAIL-R1, TRAIL-R2, and Fas Death Receptors Cell Surface Expression Are Not Modified by Lowbush Blueberry Pcys Treatment on SW480 Nor SW620 Cell Lines

According to the membrane disturbance activities observed on human monocytic cells and according to the fact that apple Pcys trigger an increase of TRAIL-R1 and TRAIL-R2 at the cell surface on both cell lines [54], we here investigated whether lowbush blueberry Pcys could induce as well an increase of the three studied death receptors, that is, TRAIL-R1, TRAIL-R2, and Fas receptors, at the cell surface of SW620 and SW480 cells.

**Figure 4:**
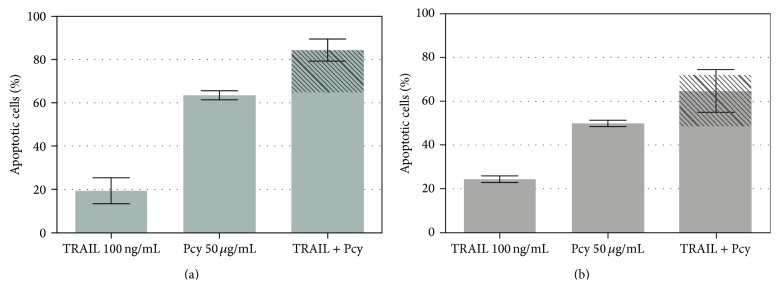
Effect of lowbush blueberry Pcy on TRAIL-mediated apoptosis in SW480 and SW620 cells. The nonsynergistical effects of TRAIL (100 ng/mL) and lowbush blueberry proanthocyanidins (Pcys) (50 *μ*g/mL) combination on apoptosis of SW620 (a) and SW480 (b) cell lines are illustrated by the dashed area reporting TRAIL alone activation and overlaid on the data obtained for TRAIL + Pcy.

**Figure 5:**
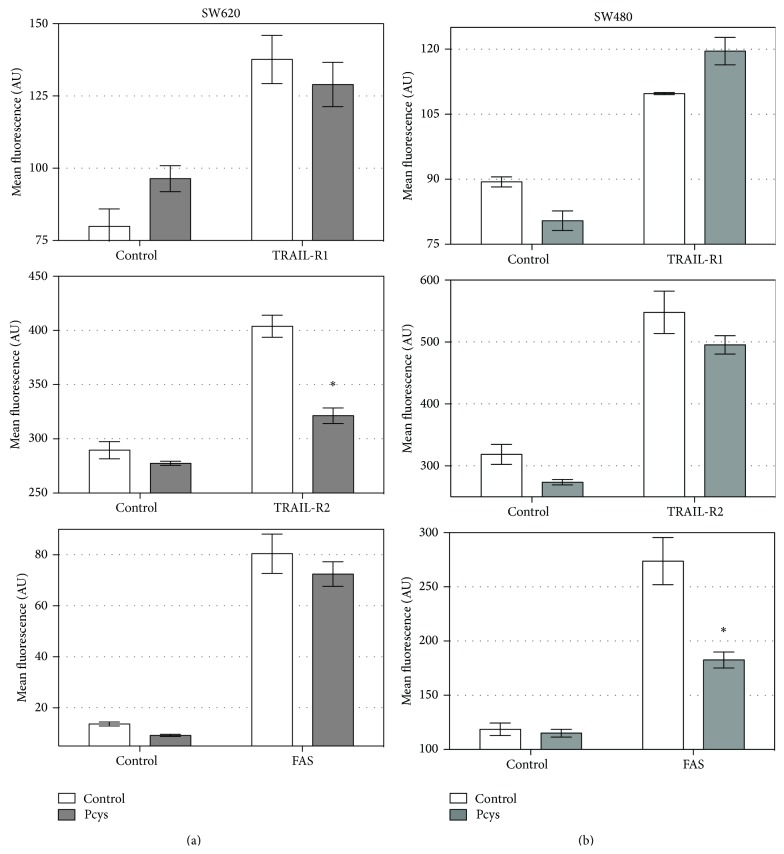
Effect of lowbush blueberry proanthocyanidins (Pcys) (50 *μ*g/mL) on the density of death receptors present at the cell surface of SW620 (a) and SW480 (b) cells. TRAIL-R1 receptor is specifically recognized by a monoclonal antibody Ac1 coupled to Alexa 488 (which emits at 525 nm) whereas TRAIL-R2 receptor is specifically recognized by a monoclonal antibody Ac2 coupled to phycoerythrin (which emits at 575 nm). Fas receptor is specifically recognized by a monoclonal antibody Ac3 coupled to phycoerythrin-Cy5 (PE-Cy5) (which emits at 670 nm). TRAIL-R1 receptor presence is materialized by the mean green fluorescence emitted by Ac1 antibody on SW620 or SW480 cell lines, while TRAIL-R2 receptor is materialized by the mean yellow fluorescence emitted by Ac2 antibody and Fas receptor is materialized by the red green fluorescence emitted by Ac3 antibody. Statistical significant differences (*n* = 3 independent experiments) based on mean fluorescence (AU) of the cell population between labeled cells in absence of proanthocyanidins and cells labeled and treated with Pcys are represented by the symbol “∗.”

Lowbush blueberry Pcys did not modify TRAIL-R1 or TRAIL-R2 (Figure 4) nor Fas receptor expression (Figure 5). Only a slight decrease could be noticed for TRAIL-R2 and Fas in SW480 cell line.

### 3.7. Lowbush Blueberry Pcys Induce Caspase 8 at 3 and 6 Hours but Not Caspase 9

**Figure 6:**
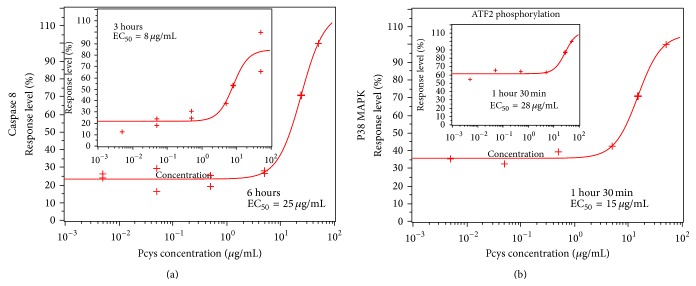
Caspase 8 and P38 MAK pathways activation by lowbush blueberry proanthocyanidins in SW620 cells. (a) Caspase 8 activation curve responses were obtained with two fluorochrome-conjugated inhibitors of caspases consisting of a fluorophore (sulforhodamine for caspase 8 and carboxyfluorescein for caspase 9), a peptide specific for the active site of a particular caspase or many caspases, and a reactive functional group (fluoromethylketone or FMK). These inhibitors are cell permeable and noncytotoxic. Once inside the cell, the caspase inhibitors bind specifically through the peptide moiety to caspases that have been activated in apoptosis, and the FMK moiety covalently links the inhibitor to the caspase. The resulting signal is proportional to the number of active caspase enzymes that are present in the cell. (b) Directly conjugated phosphospecific antibodies were used to monitor the activation of P38 MAK and ATF2 pathways. All signals were monitored by capillary flow cytometry as described under M&M.

After 24 hours, apple Pcys were shown to trigger activation of caspase 8 in both cell lines, but only caspase 9 in SW620 cells [54]. After 48 hours, the two caspases were fully activated. Our results here emphasize the fact that lowbush blueberry Pcys are more potent as they significantly activate both caspases 8 and 9 in SW620 (a) and SW480 (b) cell lines (Figure 6) after 24 and 48 hours. As for apple Pcys, shorter incubation times (i.e., 3 and 6 hours) resulted in caspase 8 activation only, highlighting the importance of the extrinsic apoptosis pathway.

### 3.8. Lowbush Blueberry Pcys Induce P38 Phosphorylation in SW620 Cells

P38 MAK is involved in regulation of Hsp27 and MAPKAP-2 and several transcription factors including ATF2, STAT1, MEF-2, and ELK-1 [55]. Monitoring the activity of cell signaling pathways (P38 and ATF2) underlined the fast activation of cell signals when apoptosis is induced by lowbush blueberry Pcys. IC_50_ value (15 *μ*g/mL) obtained for P38 phosphorylation was equivalent to the value assessed for caspase 8 activation after 3 hours (8 *μ*g/mL) showing a nice correlation between the two events.

## 4. Discussion

Pcy chemopreventive effects on CRC remain less studied as those induced by other polyphenols [56, 57]. One explanation could be that Pcy extraction is not an easy task [58, 59]. Hence, the extractable Pcys do not exactly reflect qualitatively nor quantitatively the total Pcys of the studied vegetal material. The activities of the extracted Pcys may not exactly reflect the whole fruit activity. The method we use here allows us to enrich our extracts in Pcy oligomers and polymers.

When we screened for proapoptotic activities, the Pcy-enriched fractions from various berries on the TRAIL-resistant SW620 cell line, two berries showed more activity: lingonberry and lowbush blueberry. It looks like the Pcy richness of an extract is not directly related to proapoptotic activity, but two criteria seem relevant: polymer concentration and polymerization degree (PD). Even if lingonberry contains more Pcys in terms of procyanidin A2 equivalents, lowbush blueberry, with more polymerized Pcys, shows stronger proapoptotic activities. In a same sense, raspberry and blackberry fractions, containing very few polymers, were less active on our cellular models. Indeed, small polymer percentages over total Pcys (0% for raspberry and 5,6% for blackberry), with low mPDs (2,1 for raspberry and 2,3 for blackberry), were already reported [28]. Such a Pcy polymers richness of lowbush blueberry was described before by several authors [28, 29].

The proapoptotic dose-response curves of lowbush blueberry and lingonberry Pcy-enriched extracts on SW480 and SW620 cell lines fit with a higher maximum proapoptotic activity for lowbush blueberry Pcy-enriched extract when compared to lingonberry and this is for both cell lines. Thus, the importance of the Pcy structures has to be emphasized in relation to their proapoptotic activities. The importance of their PD on antiproliferative activities was described earlier for grape seed and pine bark [60], apple [35], and American wild blueberry (*Vaccinium angustifolium*) [61]. If, on one hand, the strength of an extract* in vitro* proapoptotic activity could be linked to its polymer proportion and to their mPD, on the other hand, their chemical structures probably play, as well, an important role in their proapoptotic activities, especially

- proportions of type A and type B boundings and their respective positions inside the Pcy molecule;
- proportions of the different possible flavan-3-ol units (e.g., (epi)catechin, (epi)gallocatechin, and (epi)afzelechin) and respective positions inside the Pcy molecule; lingonberry (*Vaccinium vitis-idaea*) contains types A and B procyanidins (catechin/epicatechin) whereas lowbush blueberry (*Vaccinium myrtillus*) contains as well type A and B prodelphinidins (gallocatechin/epigallocatechin) [28–30].

All these parameters will influence the Pcy tridimensional structure and therefore their interaction with cellular elements (receptors, membranes), conditioning biological activities [62–64]. This could be one of the reasons why the most active Pcy extract is the one obtained from lowbush blueberry which, among all tested fruits, presents the higher polymer rate.

TRAIL and related signalization pathways via its receptors TRAIL-R1 and TRAIL-R2 were monitored in view of the capacity of lowbush blueberry Pcy-enriched extract to induce a very strong apoptosis (92–95% at 75 *μ*g/mL) on both cell lines, which notably differ on their TRAIL sensitivity. The mechanism of action could involve TRAIL, inducing suppression of TRAIL resistance in the SW620 cells. The Fas receptor, representative of the TNF superfamily death receptors, was monitored in parallel, as some phytoconstituents were previously described as being able to induce cells apoptosis via extrinsic pathway activation and increase of death receptors number at the cell surface, whether it was for TRAIL-R1 and R2, with sensitization to TRAIL-induced apoptosis [37, 65, 66] or Fas [67–69].

In our hands, lowbush blueberry Pcys were not capable of sensitizing neither SW480 nor SW620 cells to commit TRAIL-induced apoptosis. Indeed, lowbush blueberry Pcys treatment did not modify the expression of TRAIL-R1 and TRAIL-R2 as well as Fas death receptors at the cell surface, and this is for both cell lines.

Unaffected death receptor's numbers at the cell surface as well as nonsensitization to TRAIL by lowbush blueberry Pcys can be explained in two different ways. Lowbush blueberry Pcy-induced apoptosis is mediated

- by TRAIL-R1, TRAIL-R2, and/or Fas receptors with an increase of their number at the cell surface but highly polymerized lowbush blueberry Pcys, by forming a coating around the cell, prevented their detection by specific antibodies,
- by other TNF superfamily death receptors inducing caspases 8 and 9 activation, for example, TNF-R1, DR3, or DR6; further experimentations to elucidate these issues were considered, that is, using fluorescent labeled Pcys to visualize any cell surface/receptor coating; however, any chemical grafting of a fluorescent entity will induce changes in the chemophysical properties of the labeled Pcys; fluorescent entities are always presenting high ring density of *π* electrons generating the fluorescence; thus, such grafted Pcys will compromise their interaction with cellular membrane not permitting to decide which explanation is the most realistic, even with confocal image based investigations.

Nevertheless, lowbush blueberry Pcys trigger apoptosis via the extrinsic pathway, and this is for both colonic cell lines. It is only after 48 hours that caspases 8 and 9 are similarly greatly activated in the two cell lines (70–80%). We know that extrinsic (caspase 8) and intrinsic (caspase 9) pathways are linked by protein Bid: activated caspase 8 splits Bid, which then later activates caspase 9. Both caspases can then be activated simultaneously only when both extrinsic and intrinsic apoptosis pathways are activated. The whole process is thus triggered by the extrinsic pathway, that is to say, from the cell membrane, consistent with the fact that Pcys (starting from trimers) are unable to enter the cell [63]. In view of the early P38 MAK pathway activation one could make a parallel to apoptosis induced by ROS entities following an oxidative stress. Then lowbush blueberry Pcys are not commonly accepted as passing passively the cellular membrane, unless one takes into account the phenomenon of “sliding through the membrane” recently stated by the group of P. Trouillas [70, 71]. These authors were interested particularly in polyphenols and other *π*-conjugated compounds. Their molecular dynamics simulations give a very good estimate of the ability of these compounds to insert into membranes. Positions and “exact” directions compounds can thus be obtained* in silico*. Such an insert into the membrane could easily activate the P38 MAK pathways and caspase 8.

The degree of polymerization of proanthocyanidins has a major impact on their fate in the body [28]. Studies have shown that the proanthocyanidins are not degraded in the stomach. If conditions are in any case not quite drastic, they are also protected by the buffering effect of the bolus [34, 72]. The proanthocyanidol polymers appear to be 10 to 100 times less well absorbed than monomers [27, 28]. Thus, the polymers are much less well absorbed through the digestive track portion, monomers, dimers, and trimers [73], due to their lower cellular absorption and their complexation with protein and luminal mucosa [27, 72, 74]. During passage through the small intestine, the proanthocyanidol polymers form complexes with proteins, starch, and digestive enzymes; these complexes are less easily digested, explaining why proanthocyanidol polymers thus reach the colon unchanged [73]. Dimers and trimers intact and undamaged trimers were detected at low levels in urine and plasma of rats after ingestion of a procyanidin-rich extract from grape seeds [75, 76], proving their limit to the systemic circulation; gastrointestinal absorption of procyanidins from the tetramer is suggested to be low or zero. In addition, human plasma levels of procyanidins are very low (nanomolar) after ingestion of cocoa [77] and grape seed [78]. Proanthocyanidins with a polymerization degree greater than or equal to 2 are not depolymerized bioavailable monomers during their passage through the gastrointestinal tract [28, 72, 73, 76].

Authors previously described over a physiologically relevant dose range (up to 50 *μ*g/mL gallic acid equivalents) that digested and fermented berry extracts demonstrated significant activities on colonocytes [79] indicating that phenolic compounds from berries, even after their passage through the gastrointestinal tract, retain biological activity and can modulate cellular processes associated with colon cancer. So once procyanidins have reached the colon, they can do their job. The enriched extract of blueberry procyanidins contains large procyanidins (trimers and beyond) that cannot enter the cells, as already demonstrated [80, 81], but may have effects on cell membranes [80, 82]. Some authors showed that the polymers are not absorbed across a monolayer of Caco-2 cells and partially adhere to the cell surface [83]. Furthermore, Maldonado-Celis showed that apple procyanidin activates the extrinsic pathway of apoptosis via membrane receptors [37].

In conclusion, Pcys beyond trimers, the major dietary Pcys, are not absorbed throughout the digestive tract, particularly due to their huge molecular weights [27, 74]. Thus, they reach the colon practically intact, where they are able to locally exert their anticancer activities on colorectal precancerous and cancerous cells [72]. Although their metabolism by the colon microflora still remains unclear [74], it is believed to play a major role in Pcys varied biological activities* in vivo* [28, 75, 84]. We estimated that a human of 60–75 kg bodyweight should ingest 40 ± 5 g/day of fresh blueberries to have a quantity of procyanidins reaching the colon equivalent to the one tested in our* in vitro* experiments. This amount seems reasonably attainable through diet.

Our present work emphasizes the potential of berries in chemoprevention, especially CRC chemoprevention, due in part to their polyphenol content and notably to their neglected Pcys. To our knowledge, this is the first report on proapoptotic activities of lowbush blueberry and lingonberry Pcys on human colorectal cell lines.

## Figures and Tables

**Table 1:** Procyanidin A2 equivalents of apple Pcys, lowbush blueberry, and lingonberry Pcy-rich extracts obtained by the BL-DMAC dosage as described under M&M.

Proanthocyanidin-rich sample	Procyanidin A2 equivalents in mg/100 g fresh fruit
Apple	72 ± 10
Lingonberry	48 ± 7
Lowbush blueberry	33 ± 5

**Table 2:** EC_50_ and maximum proapoptotic activity, expressed in percentage of apoptotic cells. Results were observed after 24 h (SW620) or 48 h (SW480) treatment with apple Pcys, lowbush blueberry, and lingonberry Pcy-rich extracts.

Fruit	Human colon cancer cell line			
	SW620	SW480	SW620	SW480
	EC_50_ in *µ*g/ml		% of apoptotic cells at 75 *µ*g/ml	
Lowbush blueberry	24.7 ± 0.1	25.2 ± 4.5	95 ± 1%	92 ± 6%
Lingonberry	24.3 ± 0.2	24.7 ± 0.1	37 ± 2%	41 ± 5%
Apple	46.0 ± 4.5	34.6 ± 0.3	64 ± 9%	55 ± 9%

**Table 3:** Cell cycle analyses on THP-1 monocytic cells. Results were observed after 6 and 24 h treatment with lowbush blueberry Pcy-rich extract (50 *µ*g/ml).

Time	Pcys	Sub G0/G1	G0/G1	S	G2/M	% ± SD
6 h	—	0.4 ± 0.2	68.8 ± 5.0	13.0 ± 2.0	17.7 ± 4.3	(*n* = 12)
	Lowbush blueberry	0.4 ± 0.2	66.3 ± 6.5	13.7 ± 1.7	19.5 ± 6.6	(*n* = 6)

24 h	—	0.7 ± 0.8	66.1 ± 6.1	17.4 ± 3.9	15.8 ± 8.3	(*n* = 14)
	Lowbush blueberry	0.6 ± 0.2	69.4 ± 3.4	13.1 ± 5.0	16.8 ± 5.8	(*n* = 6)

## Abbreviations

DMAC: * p*-Dimethylaminocinnamaldehyde
Pcy: Proanthocyanidin
PD: Polymerization degree
TRAIL: TNF-related apoptosis-inducing ligand.
